# Kinematic Differences Based on Shooting Proficiency and Distance in Female Basketball Players

**DOI:** 10.3390/jfmk8030129

**Published:** 2023-09-05

**Authors:** Dimitrije Cabarkapa, Damjana V. Cabarkapa, Nicolas M. Philipp, Chloe A. Myers, Shay M. Whiting, Grant T. Jones, Andrew C. Fry

**Affiliations:** Jayhawk Athletic Performance Laboratory–Wu Tsai Human Performance Alliance, Department of Health, Sport and Exercise Sciences, University of Kansas, Lawrence, KS 66045, USA; dcabarkapa98@gmail.com (D.V.C.); nicophilipp@ku.edu (N.M.P.); chloe.myers@burrell.edu (C.A.M.); whitingsm99@gmail.com (S.M.W.); gtjones02@ku.edu (G.T.J.); acfry@ku.edu (A.C.F.)

**Keywords:** biomechanics, video analysis, distance, two-point, three-point, jump-shot, coaching

## Abstract

The purpose of the present study was to examine differences in kinematic characteristics between (a) proficient and non-proficient two-point and three-point shooters, (b) made and missed two-point and three-point shots within a proficient group of shooters, and (c) shots attempted from two-point and three-point shooting distances. Eighteen recreationally active females with previous basketball playing experience attempted 10 two-point (5.10 m) and 10 three-point shots (6.32 m) while facing directly to the basket. To eliminate the possible influence of fatigue, each shot was separated by a 5–10 s rest interval. Participants who made ≥50% of their two-point and ≥40% of their three-point shooting attempts were classified as proficient. A high-definition video camera recording at 30 fps and video analysis software (Kinovea) were used to obtain the kinematic variables of interest during both the preparatory phase (PP) and release phase (RP) of the shooting motion. The results indicate that proficient two-point shooters attained less hip and shoulder flexion during the PP and had greater release height and vertical displacement during the RP. Hip angle differentiated made from missed two-point shots within the proficient group of shooters, with made shots being depicted by less hip flexion. Significantly greater vertical displacement was observed in proficient three-point shooters during the RP. Additionally, the greater elbow and release angles separated made from missed three-point shots within the proficient group of shooters. In response to an increase in shooting distance, hip, knee, ankle, and shoulder angles during the PP all decreased. Moreover, an increase in shooting distance caused a decrease in release angle and an increase in vertical displacement during the RP, while the relative release height remained unchanged.

## 1. Introduction

Shooting efficiency has been shown to be one of the key performance parameters differentiating winning from losing game outcomes in basketball, ranging from amateur to professional levels of competition [1,2,3,4,5,6,7]. In a recently published study examining game-related statistics within a cohort of elite women’s basketball players, Madarme [8] found that successful two-point field goals discriminated winning from losing teams during balanced games played on Under-19 level of competition (i.e., a final score differential <16 points). Also, the same variable was found to be among the most powerful performance metrics capable of classifying starters from non-starters in the Women’s National Basketball Association league [9]. Therefore, it is understandable why a considerable emphasis is placed on developing and/or optimizing individual players’ shooting efficiency as a crucial factor that can ultimately help a team secure the desired game outcome.

A considerable amount of scientific literature has been focused on analyzing various biomechanical characteristics related to optimizing shooting performance in male basketball players, including free-throw, two-point, and three-point shooting motions [10,11,12,13,14,15,16]. For example, when examining a group of college-age males during the learning process, Ammar et al. [10] found that knee angle kinematics were highly correlated with free-throw shooting performance. A lower knee flexion (i.e., less knee bend) during the preparatory phase and greater knee extension during the release phase of the free-throw shooting motion were positively associated with a greater number of made baskets [10]. Further, it has been found that proficient two-point shooters demonstrated higher elbow placement and greater elbow flexion during the preparatory phase of the shooting motion, while proficient three-point shooters attained greater vertical displacement and shoulder release angle during the release phase of the shooting motion [13]. However, the amount of research reports focused on examining the aforementioned biomechanical parameters within female athletes is sparse [17,18,19]. Hudson [17] found that highly skilled female shooters (i.e., national team players) attained a significantly greater ball release height when compared to moderate and low-skill shooters. On the other hand, Vencurik et al. [18] found no difference in shoulder angle at ball release between successful and unsuccessful two-point and three-point shooting attempts in Under-16 and Under-18 female basketball players. In addition, the authors in the same investigation noted that female basketball players displayed different shooting technique than male basketball players (e.g., lower shoulder angle at ball release, smaller entry angle, larger center of mass displacement in the horizontal direction) [18], further emphasizing the importance of studying the biomechanics of shooting motion within this group of athletes.

Besides being solely focused on examining differences between proficient and non-proficient shooters, the impact of distance on biomechanical parameters of shooting motion cannot be disregarded [20]. Previous research has shown that shooting efficiency is inversely associated with an increase in shooting distance [19,21]. A recently published study on professional male basketball players found that three-point shooting motions required lower elbow positioning, influenced by greater knee and hip flexion, when compared to free-throw and two-point shooting motions [14]. Further, the release angle and ball release height tend to decrease with an increase in the shooting distance [22]. When studying similar kinematic parameters within a group of professional female basketball players, Elliott and White [23] found that three-point shots required less shoulder and wrist flexion than two-point shots. Additionally, the time spent in the air for three-point shoots was considerably greater, while no statistically significant differences were observed in knee and hip angles during the release phase of the shooting motion [23]. Still, the issue pertaining to the lack of scientific literature focused on examining this area of female athlete performance remains present.

Thus, to bridge a gap in the scientific literature and provide a deeper understanding of the biomechanics of some of the most commonly implemented shooting motions, the purpose of the present study was to examine differences in kinematic characteristics between (a) proficient and non-proficient two-point and three-point shooters, (b) made vs. missed shots within a group of proficient shooters, and (c) shots attempted from two-point and three-point shooting distances in female basketball players.

## 2. Materials and Methods

### 2.1. Participants

Eighteen recreationally active females (age = 22.9 ± 2.9 years, height = 170.2 ± 7.5 cm, body mass = 65.2 ± 8.6 kg; body mass index = 22.7 ± 2.9 kg/m^2^) with ≥4 years of basketball playing experience (e.g., high school, college) volunteered to participate in this study. All participants reported no current and/or previous musculoskeletal injuries that could impair the full joint range of motion and participated 1–2 times per week in basketball training activities. Additionally, all participants were instructed to abstain from strenuous exercise (e.g., resistance training) >48 h prior to the start of the testing session. The testing procedures performed in this study were previously approved by the University of Kansas Institutional Review Board, and all athletes signed an informed consent document.

### 2.2. Procedures

**Figure 1:**
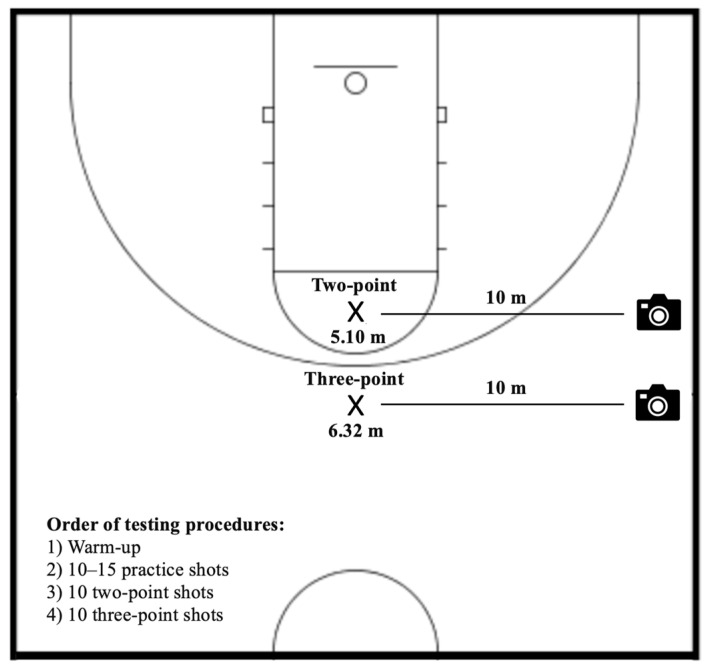
Graphical representation of the testing procedures.

Upon arrival at the laboratory, participants were familiarized with the testing procedures and proceeded with a standardized warm-up protocol consisting of dynamic stretching exercises (e.g., high knees, A-skips, walking lunges, quad pulls, butt kicks) and 10–15 practice shots from self-selected distances. Then, while facing directly to the basket, each participant attempted 10 two-point (5.10 m) and 10 three-point shots (6.32 m). A high-definition video camera (Canon PowerShot SX530, Canon Inc., Tokyo, Japan) sampling at 30 fps, positioned 10 m away perpendicular to the participant’s shooting location, was used to record each shooting motion from a sagittal point of view (Figure 1). Video analysis software (Kinovea, Version 0.8.27) was used to analyze the two-dimensional kinematic variables of interest. To eliminate the possible influence of fatigue, each shot was separated by a 5–10 s rest interval, and a research assistant was present throughout all testing procedures to complete rebounding and passing tasks. The basketball goal height (3.05 m) and size (0.72 m) corresponded to the women’s National Collegiate Athletic Association (NCAA) regulation standards. In addition, to minimize any kind of possible distractions, participants individually performed all testing procedures.

### 2.3. Variables

**Figure 2:**
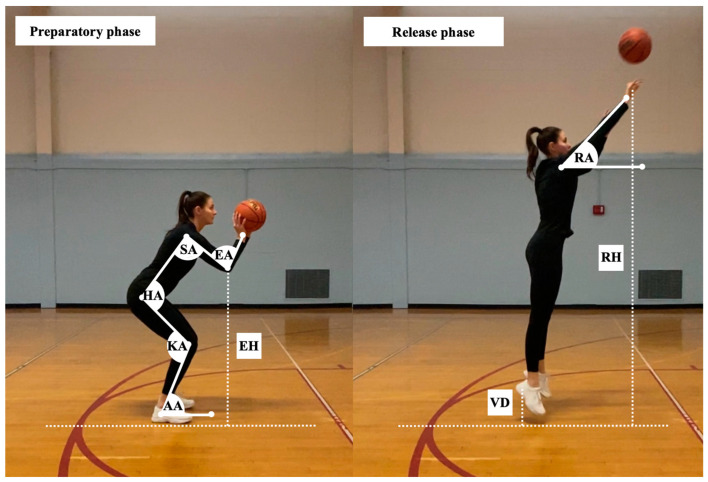
Graphical representation of dependent variables examined in the present study. Knee angle (KA), hip angle (HA), elbow angle (EA), ankle angle (AA), shoulder angle (SA), elbow height (EH), release angle (RA), release height (RH), vertical displacement (VD).

The selection of kinematic parameters examined in the present study was based on previous research reports [11,12,13,14,20,23,24]. The following variables were examined during the preparatory phase of the shooting motion (i.e., initial concentric phase): knee angle (i.e., internal angle between the thigh and shank), hip angle (i.e., internal angle between the torso and the thigh), ankle angle (i.e., relative angle between the shank and the ground), elbow angle (i.e., internal angle between the upper arm and forearm), shoulder angle (i.e., relative angle between the upper arm and torso), and elbow height (i.e., perpendicular distance between the olecranon process and the ground relative to the participant’s height). The following variables were examined during the release phase of the shooting motion (i.e., timepoint when the ball left the shooter’s hand): release angle (i.e., relative angle between the fully extended upper limb and a line parallel to the ground), release height (i.e., perpendicular distance between the center of the hand and the ground relative to the participant’s height), and vertical displacement (i.e., perpendicular distance between the calcaneus and the ground). See Figure 2.

### 2.4. Statistical Analysis

The sample size in the present study was based on previously published research reports as well as pilot data collected in our lab [10,11,12,23]. The Shapiro–Wilk test was used to examine if the assumption of normality was violated. When examining the differences in kinematic variables between proficient and non-proficient two-point and three-point shooters as well as between the two shooting distances, the mean value across 10 shots attempted by each participant was used for analysis purposes. Since all dependent variables met the assumption of normality, independent t-tests were used to examine statistically significant differences in the kinematic characteristics between proficient and non-proficient shooters, separately for each shooting distance [14,22]. Participants who made ≥50% of their two-point (*n* = 10) and ≥40% of their three-point shooting attempts (*n* = 8) were classified as proficient shooters, and the ones who failed to meet the aforementioned criteria were classified as non-proficient [13]. In addition, paired sample t-tests were used to examine differences in the kinematic variables of interest between two-point and three-point shooting distances (5.10 vs. 6.32 m).

On the other hand, when examining the differences between made and missed shots within the proficient group of shooters, all variables violated the assumption of normality, except for the hip angle, shoulder angle, elbow height, and release height for three-point shooting motion. Therefore, Mann–Whitney U-tests were used to examine statistically significant differences in the kinematic characteristics between made and missed shots for non-normally distributed variables and independent t-tests for normally distributed variables. The effect size of these differences was interpreted based on Cohen’s [25] recommendations: 0.2-small effect, 0.5-moderate effect, and >0.8 large effect. Statistical significance was set a priori to *p* < 0.05. All statistical analyses were completed with SPSS (V.26.0; IBM Corp., Armonk, NY, USA).

## 3. Results

The average shooting percentage of proficient and non-proficient two-point and three-point shooters was 64.0 ± 9.7 and 32.5 ± 7.1, and 55.7 ± 9.8 and 23.0 ± 9.5%, respectively. Proficient two-point shooters demonstrated greater hip and lower shoulder angle values during the preparatory phase of the shooting motion and notably greater release height and vertical displacement at the timepoint of the ball release. No statistically significant differences were observed for elbow height and knee, elbow, ankle, and release angles (Table 1). On the other hand, proficient three-point shooters attained considerably greater vertical displacement at the timepoint of the ball release, while no statistically significant differences were noted for any other kinematic variables of interest (Table 2).

When examining the differences in kinematic characteristics between made and missed two-point and three-point shots within a proficient group of shooters, the only statistically significant difference was observed for the hip angle. Made two-point shots were characterized by less flexion in the hip joint (Table 3). Moreover, the only statistically significant differences found between made and missed three-point shots within the proficient group of shooters were in the elbow angle and release angle. The elbow angle was smaller and the release angle was greater for made when compared to missed three-point shots (Table 4).

In addition, statistically significant differences between two-point and three-point shooting motions were observed in all kinematic variables examined in the present study, except for the elbow angle and release height. Smaller knee, hip, ankle, and shoulder angles and lower elbow positioning were found during the preparatory phase for three-point when compared to two-point shooting motion. Moreover, three-point shots were characterized by a notably lower release angle and greater vertical displacement (Table 5).

## 4. Discussion

The findings of the present study reveal notable differences in jump shot biomechanics pertaining to shooting proficiency, made vs. missed outcome of the shooting motion, as well as the impact of shooting distance. To the best of our knowledge, this is the first study that implemented a comprehensive analysis approach to examine the influence of previously mentioned factors on shooting characters in female basketball players.

### 4.1. Two-Point–Preparatory Phase

During the preparatory phase of the shooting motion, proficient two-point shooters attained less hip and shoulder flexion when compared to non-proficient shooters, while no significant differences were observed in the knee, elbow, and ankle joints. Also, it is interesting to note that no difference in the elbow height was observed between the two groups of shooters. These findings are contradictory to the observations made by Cabarkapa et al. [13] in a recently published study, which focused on examining the biomechanical characteristics of jump shooting motions in college-age male basketball players. While the magnitude of the hip angle remained unchanged, the authors found that proficient male two-point shooters attained greater elbow height and shoulder flexion during the preparatory phase of the shooting motion than non-proficient shooters [13]. Usually, it is expected that keeping the torso in a more erect position (i.e., less hip flexion) would ultimately result in greater elbow height, considering that no other changes in kinematic variables are present. However, our results reveal that proficient female shooters during the preparatory phase of the shooting motion had greater shoulder flexion. Hence, it is likely that this kinematic alteration counterweighed the expected changes in the elbow height. While the aforementioned discrepancies in these findings may be gender-specific, further research is warranted to examine if the observed differences impact the kinetic parameters of two-point shooting motions (e.g., peak concentric force, impulse). In addition, it should be noted that hip angle (i.e., trunk forward lean) was the only statistically significant variable differentiating between made and missed shots within the proficient group of shooters, with made shots being depicted by a ~4.0 degrees greater hip extension. Previous research has suggested that it is beneficial to keep the trunk in a near vertical position once the shooter has left the ground (e.g., transition phase) [24]. Despite being assessed at different timepoints of the shooting motion, these findings offer further support to the observations made in the present study. It can be assumed that less forward lean (i.e., less hip flexion) during the preparatory phase makes it easier for proficient shooters to attain near vertical torso alignment during the transition phase of the shooting motion and ultimately results in increased chances of securing the desired outcome.

### 4.2. Two-Point–Release Phase

When examining the release phase of the two-point shooting motion, proficient shooters attained a considerably greater relative release height and vertical displacement when compared to non-proficient shooters, while no difference was observed in the release angle. Although focused on examining the biomechanical characteristics of the free-throw shooting motion, Hudson’s [17] study yielded similar conclusions. High-skill free-throw shooters demonstrated greater release heights than moderate and low-skill shooters, regardless of the individual’s stature [17]. This specific parameter (i.e., release height) has been shown to be of critical importance for the successful outcome of the shooting motion [20,24]. A greater release height requires a lower launching velocity, which ultimately increases the margin of error and the likelihood of a successful shooting outcome [20,26]. Thus, players are often encouraged to jump and release the ball close to the highest point of the shooting motion [24]. Based on our findings, we can conclude that proficient two-point shooters implemented the aforementioned coaching cues related to the successful execution of the mid-range jump-shooting motion. On the other hand, despite not reaching the level of statistical significance, proficient two-point shooters tended to attain ~7.0 degrees greater release angle. Previous research reports have suggested that the release angle is positively associated with the entry angle of the ball through the rim [20,27]. A greater entry angle allows the shooter to use a larger area of the basket (i.e., rim diameter) [20,26,28]. While further research pertaining to the basketball shooting trajectory is needed, our findings further support the benefit of a greater release angle. In addition, it is important to note that our results revealed no difference between made and missed two-point shots during the release phase of the shooting motion within the proficient group of shooters, including trivial effect sizes. Rather than implying that these kinematic variables are irrelevant to the success of the shooting motion, we can assume that proficient shooters have already met these kinematic requirements and that there may be other factors (e.g., hip angle) that have a greater impact on the success of the two-point shooting motion.

### 4.3. Three-Point–Preparatory Phase

No statistically significant differences were observed in the present study between the proficient and non-proficient three-point shooters during the preparatory phase of the shooting motion. Although not statistically significant, moderate effect sizes were observed for the elbow and shoulder angles, with both values being ~5.8 degrees greater within a group of non-proficient shooters. Still, these findings suggest that proficient shooters tend to have greater flexion in the elbow and shoulder joints during the preparatory phase of the shooting motion. In a recently published study, Cabarkapa et al. [13] obtained analogous findings pertaining to a greater elbow flexion being associated with superior shooting proficiency within a cohort of college-age male basketball players. However, the authors of the same study did not detect notable changes in shoulder angle values [13]. As previously noted, this might be another gender-specific difference, but it can also be attributed to individual differences in shooting form that require further research. Likewise, when examining a cohort of elite female basketball players, Elliott and White [23] found similar elbow angle magnitudes during the preparatory phase of the shooting motion (i.e., crouch position). Specifically, the elbow angle was one of the two key kinematic variables differentiating between made and missed baskets within the proficient group of three-point shooters. Although being close to reaching the level of statistical significance, a similar trend has been observed in male counterparts [28]. The median was ~2.0 degrees lower for made compared to missed three-point shots [28]. In the present study, focusing on college-age female basketball players, made shots were characterized by ~6.0 degrees greater elbow flexion.

### 4.4. Three-Point–Release Phase

Another interesting observation made in the present study is related to the significantly greater vertical displacement observed in proficient when compared to non-proficient three-point shooters during the release phase of the shooting motion. Proficient shooters attained ~4.7 cm greater jump heights than non-proficient shooters. This was the only statistically significant kinematic variable capable of differentiating between these two groups based on their shooting proficiency level. As previously indicated, the ability to achieve a greater release height has been considered a performance trait of skilled shooters [24]. Successful jump shots have been associated with minimizing horizontal body movements and maximizing vertical jump displacement with near-vertical trunk alignment, which ultimately allows players to shoot with a greater margin of error [24]. However, despite a significant increase in vertical displacement, the release height remained relatively similar between proficient and non-proficient three-point shooters. This observation seems to disagree with our findings pertaining to the two-point shooting motion, as vertical displacement and release height seemed to be directly related. While these differences will be discussed in more detail in the following paragraph, the release angle is expected to decrease in response to an increase in shooting distance [15,20,22]. Therefore, we can assume that no statistically significant difference in the release height may be attributed to the aforementioned decrease in the angle of the ball release. Further, the release angle was shown to be the only statistically significant kinematic variable that differentiated between made and missed three-point shots within the proficient group of shooters, with release angles for made shots being ~4.0 degrees greater in magnitude.

### 4.5. Shooting Distance–Preparatory Phase

As a result of an increase in the shooting distance (i.e., two-point vs. three-point), multiple kinematic variables of interest have been considerably affected. During the preparatory phase of the shooting motion, hip, knee, ankle, and shoulder angles all decreased in magnitude for three-point compared to two-point shots. These kinematic changes resulted in lower elbow positioning, while the elbow angle remained unchanged. Similar observations were made by Cabarkapa et al. [14] when examining professional male basketball players. An increase in shooting distance required greater flexion in the knee and hip joints during the preparatory phase of the shooting motion [14]. Additionally, Elliott and White [23] found that angular displacement notably decreased with an increase in the shooting distance in elite female basketball players. Based on previous research, the aforementioned biomechanical alterations in shooting form observed in the present study are expected [14,20,22]. As the shooter moves further away from the basket, they need to readjust the coordination of multiple body segments in order to generate more force to propel the ball toward the basket [14,20,22].

### 4.6. Shooting Distance–Release Phase

In a similar manner, an increase in shooting distance (i.e., two-point vs. three-point) resulted in a decrease in the release angle and an increase in vertical displacement during the release phase of the shooting motion, while the release height remained unchanged. As previously mentioned, it is likely that a decrease in the release angle by ~2.1 degrees, accompanied by an increase in vertical displacement by ~3.2 cm, offset the decrease in release height. Our findings seem to align with previous research reports examining the impact of distance on biomechanical parameters of jump shooting motions [22,23]. Elliott and White [23] found that shooters spent a significantly greater amount of time in the air when performing three-point than two-point jump shots. Further, Okazaki and Rodacki [23] found that the release angle decreased when shots were performed at mid-range compared to near-basket shooting distances. Additionally, the same group of authors found no statistically significant difference in the release height between two-point and three-point shots, which is similar to the findings observed in the present study [23].

### 4.7. Limitations

While attempting to address underexamined aspects of the shooting performance in female basketball players, this study is not without limitations. The shooting procedures were non-fatiguing and they were performed in a laboratory-based setting without the presence of a defender and an audience, which could have had an impact on the shooting accuracy. Additionally, the kinetic characteristics and kinematic chaining (i.e., a combination of movements in the joints linked together) of two-point and three-point shooting motions as well as how they change in response to an increase in the shooting distance were not examined in the present study and warrant further investigation. Lastly, further research should also examine if these findings are applicable to female basketball players competing on other competitive levels (e.g., junior, professional).

## 5. Conclusions

The findings of the present study reveal notable differences in jump shot biomechanical characteristics pertaining to shooting proficiency, made vs. missed outcomes of the shooting motion, as well as the impact of shooting distance.

Proficient two-point shooters attained less hip and shoulder flexion during the preparatory phase of the shooting motion and had greater release height and vertical displacement during the release phase of the shooting motion when compared to non-proficient shooters. Hip angle was the only variable capable of differentiating made from missed two-point shots within the proficient group of shooters, with made shots being depicted by less hip flexion.

While no statistically significant differences were observed between the proficient and non-proficient three-point shooters during the preparatory phase of the shooting motion, a significantly greater vertical displacement was observed in proficient three-point shooters during the release phase of the shooting motion. Also, greater elbow and release angles were the only two variables capable of separating between made and missed three-point shots within the proficient group of shooters.

In response to an increase in the shooting distance, hip, knee, ankle, and shoulder angles during the preparatory phase of the shooting motion all decreased. Moreover, an increase in shooting distance caused a decrease in the release angle and an increase in vertical displacement during the release phase of the shooting motion, while the relative release height remained unchanged.

Overall, these findings could provide coaches and players with guidelines on which adjustments in shooting form need to be made during the learning process to elicit improvements in shooting accuracy and ultimately optimize on-court basketball performance.

## Figures and Tables

**Table 1:** Descriptive statistics, mean (standard deviation), *p*-values, and effect sizes (ES) for differences in kinematic characteristics between proficient (≥50%) and non-proficient (<50%) two-point shooters.

Variable	Proficient	Non-Proficient	*p*-Value	ES
Knee angle [deg]	115.6 (10.9)	113.8 (6.3)	0.672	0.196
Hip angle [deg] *	136.9 (6.2)	128.6 (10.0)	0.048	1.027
Elbow angle [deg]	50.4 (13.4)	54.6 (9.4)	0.460	0.355
Ankle angle [deg]	57.7 (8.9)	57.7 (4.2)	0.997	0.000
Shoulder angle [deg] *	52.3 (11.6)	63.8 (6.7)	0.025	1.178
Elbow height [ratio]	0.61 (0.04)	0.59 (0.05)	0.418	0.448
Release angle [deg]	44.7 (10.8)	37.7 (8.0)	0.147	0.723
Release height [ratio] *	1.40 (0.10)	1.28 (0.07)	0.010	1.361
Vertical displacement [cm] *	24.3 (3.8)	19.9 (5.0)	0.047	1.008

Note: * significant difference between proficient and non-proficient shooters (*p* < 0.05).

**Table 2:** Descriptive statistics, mean (standard deviation), *p*-values, and effect sizes (ES) for differences in kinematics characteristics between proficient (≥40%) and non-proficient (<40%) three-point shooters.

Variable	Proficient	Non-Proficient	*p*-Value	ES
Knee angle [deg]	109.6 (5.8)	109.2 (11.6)	0.925	0.042
Hip angle [deg]	128.0 (11.5)	124.9 (14.4)	0.629	0.235
Elbow angle [deg]	49.1 (11.5)	55.0 (12.2)	0.314	0.496
Ankle angle [deg]	54.9 (4.7)	55.8 (6.9)	0.761	0.149
Shoulder angle [deg]	50.9 (9.5)	56.8 (11.3)	0.253	0.559
Elbow height [ratio]	0.57 (0.04)	0.56 (0.05)	0.528	0.218
Release angle [deg]	42.7 (11.6)	37.2 (10.1)	0.301	0.510
Release height [ratio]	1.36 (0.09)	1.33 (0.12)	0.607	0.278
Vertical displacement [cm] *	28.1 (4.2)	23.4 (3.2)	0.015	1.280

Note: * significant difference between proficient and non-proficient shooters (*p* < 0.05).

**Table 3:** Descriptive statistics, median (interquartile range), *p*-values, and effect sizes (ES) for differences in kinematics characteristics between made and missed two-point shots within a proficient group of shooters.

Variable	Made	Missed	*p*-Value	ES
Knee angle [deg]	117.5 (9.0)	114.0 (15.8)	0.115	0.158
Hip angle [deg] *	137.0 (10.3)	133.0 (9.0)	0.048	0.198
Elbow angle [deg]	52.0 (23.2)	53.5 (24.3)	0.918	0.010
Ankle angle [deg]	55.0 (9.3)	56.0 (10.3)	0.434	0.078
Shoulder angle [deg]	51.5 (18.5)	48.5 (12.5)	0.439	0.078
Elbow height [ratio]	0.61 (0.06)	0.59 (0.06)	0.108	0.161
Release angle [deg]	43.0 (17.3)	43.5 (21.5)	0.502	0.067
Release height [ratio]	1.39 (0.07)	1.39 (0.15)	0.938	0.008
Vertical displacement [cm]	23.1 (4.2)	23.4 (6.2)	0.251	0.115

Note: * significant difference between made and missed shoots (*p* < 0.05).

**Table 4:** Descriptive statistics, median (interquartile range) or mean (standard deviation), *p*-values, and effect sizes (ES) for differences in kinematics characteristics between made and missed three-point shots within a proficient group of shooters.

Variable	Made	Missed	*p*-Value	ES
Knee angle [deg]	110.0 (20.0)	111.0 (10.0)	0.696	0.044
Hip angle [deg] ^#^	129.3 (13.1)	126.8 (9.6)	0.332	0.262
Elbow angle [deg] *	47.0 (15.0)	53.0 (22.0)	0.013	0.277
Ankle angle [deg]	56.0 (7.0)	55.0 (8.5)	0.218	0.138
Shoulder angle [deg] ^#^	51.6 (11.7)	48.3 (11.1)	0.193	0.290
Elbow height [ratio] ^#^	0.58 (0.04)	0.57 (0.04)	0.343	0.250
Release angle [deg] *	42.0 (18.0)	38.0 (17.5)	0.020	0.260
Release height [ratio] ^#^	1.38 (0.08)	1.35 (0.10)	0.158	0.330
Vertical displacement [cm]	28.8 (3.9)	28.8 (7.3)	0.851	0.021

Note: * significant difference between made and missed shots (*p* < 0.05); ^#^ denotes mean (standard deviation).

**Table 5:** Descriptive statistics, mean (standard deviation), *p*-values, and effect sizes (ES) for differences in kinematics characteristics between two-point and three-point shooting motions.

Variable	Two-Point	Three-Point	*p*-Value	ES
Knee angle [deg] *	114.8 (8.9)	109.4 (9.2)	<0.001	0.597
Hip angle [deg] *	133.2 (8.9)	126.3 (12.9)	<0.001	0.622
Elbow angle [deg]	52.3 (11.7)	52.4 (11.9)	0.854	0.008
Ankle angle [deg] *	57.7 (7.0)	55.4 (5.9)	0.021	0.355
Shoulder angle [deg] *	57.4 (11.1)	54.2 (10.6)	0.006	0.322
Elbow height [ratio] *	0.60 (0.04)	0.56 (0.04)	<0.001	1.000
Release angle [deg] *	41.7 (10.0)	39.6 (10.8)	0.003	0.202
Release height [ratio]	1.35 (0.10)	1.34 (0.11)	0.969	0.095
Vertical displacement [cm] *	22.3 (4.8)	25.5 (4.3)	<0.001	0.702

Note: * significant difference between two-point and three-point shots (*p* < 0.05).

## Data Availability

The data presented in this study are available on request from the corresponding author.
